# Functional Heterogeneity of Protein Kinase A Activation in Multipotent Stromal Cells

**DOI:** 10.3390/ijms21124442

**Published:** 2020-06-22

**Authors:** Pyotr A. Tyurin-Kuzmin, Maxim N. Karagyaur, Konstantin Yu. Kulebyakin, Daniyar T. Dyikanov, Vadim I. Chechekhin, Anastasiya M. Ivanova, Mariya N. Skryabina, Mikhail S. Arbatskiy, Veronika Yu. Sysoeva, Natalia I. Kalinina, Vsevolod A. Tkachuk

**Affiliations:** Department of Biochemistry and Molecular Medicine, Faculty of Medicine, Lomonosov Moscow State University, 119991 Moscow, Russia; Darth_max@mail.ru (M.N.K.); konstantin-kuleb@mail.ru (K.Y.K.); danidy@inbox.ru (D.T.D.); v-chech@mail.ru (V.I.C.); ivanovanastasia14@gmail.com (A.M.I.); skrebbka@gmail.com (M.N.S.); algenubi81@mail.ru (M.S.A.); veroniks@mail.ru (V.Y.S.); n_i_kalinina@mail.ru (N.I.K.); tkachuk@fbm.msu.ru (V.A.T.)

**Keywords:** multipotent stromal cells, mesenchymal stem/stromal cells, protein kinase A, functional heterogeneity, single cell analysis, intracellular signaling, PKA-Spark biosensor

## Abstract

Multipotent stromal cells (MSC) demonstrate remarkable functional heterogeneity; however, its molecular mechanisms remain largely obscure. In this study, we explored MSC response to hormones, which activate Gs-protein / cyclic AMP (cAMP) / protein kinase A (PKA) dependent signaling, at the single cell level using genetically encoded biosensor PKA-Spark. For the first time, we demonstrated that about half of cultured MSCs are not able to activate the cAMP/PKA pathway, possibly due to the limited availability of adenylyl cyclases. Using this approach, we showed that MSC subpopulations responding to various hormones largely overlapped, and the share of responding cells did not exceed 40%. Using clonal analysis, we showed that signaling heterogeneity of MSC could be formed de novo within 2 weeks.

## 1. Introduction

Multipotent stromal cells (MSC) play a pivotal role in maintenance of tissue integrity and regeneration, especially in skeletal, connective, and epithelial tissues [1,2]. Despite similar morphology and immunophenotype, these cells demonstrate remarkable functional heterogeneity in terms of differentiation abilities and clonogenic capacities. Thus, recent findings obtained using single-cell RNAseq technologies identified about 10 distinct MSC subpopulations with different differentiation and secretion abilities [3,4]. MSCs are regulated by a number of endocrine and paracrine signals, many of which activate G-protein coupled receptors (GPCRs), such as acetylcholine [5], adenosine [6], angiotensin II [7], angiotensin 1-7 [7], ATP [8], dopamine, GABA [9], glutamate [10], histamine [11], noradrenaline [12], serotonin [13], and sphingosine-1-phosphate [14].

Previously, using single-cell signaling analysis of Ca^2+^-dependent intracellular signaling in living cells, we demonstrated that MSCs functional heterogeneity could be due to different ability to initiate G_q_/calcium-dependent cascades in response to hormones, such as noradrenaline and angiotensin II [7,10]. Moreover, Ca^2+^-dependent functional activity of MSC is regulated by cyclic AMP (cAMP)/protein kinase A (PKA)-dependent signaling activated by β-adrenoceptors [15]. cAMP/PKA signaling pathway also regulates pivotal functions of MSC, such as adipogenic differentiation [16] or regulation of resident stem cells [17]. 

However, the activation of cAMP/PKA pathways in response to hormones in living MSCs was not analyzed yet. This was due to the lack of a convenient and reliable research tool for single-cell registration of cAMP and/or PKA activation in living cells in real time. Recently, a genetically-encoded biosensor of PKA activity was developed [18]. This biosensor, named PKA-Spark, consists of polypeptide forming homo-oligomeric coiled-coil, consensus PKA substrate sequence or specific phosphopeptide-binding domain, and green fluorescent protein (GFP) as a readout. PKA-Spark evenly distributes through the cytoplasm and precipitates after PKA-dependent phosphorylation of PKA substrate motif. As a result, it forms intensively fluorescent droplets or aggregates. These droplets are accessible to protein phosphatases; thus, their forming is reversible [18]. In this study, we explored the activation of G_s_/cAMP/PKA signaling in response to several hormones in living MSCs at the single cell level using PKA-Spark biosensor. For the first time, we demonstrated that about half of cultured MSCs are not able to activate the cAMP/PKA pathway, possibly due to the limited availability of adenylyl cyclases. Using this approach, we showed that MSC subpopulations responding to various hormones largely overlapped, and the share of responding cells did not exceed 40%. Using clonal analysis, we showed that signaling heterogeneity of MSC could be formed de novo within 2 weeks. 

## 2. Results

### 2.1. MSCs Subpopulations Demonstrate Distinct PKA Activation 

**Figure 1:**
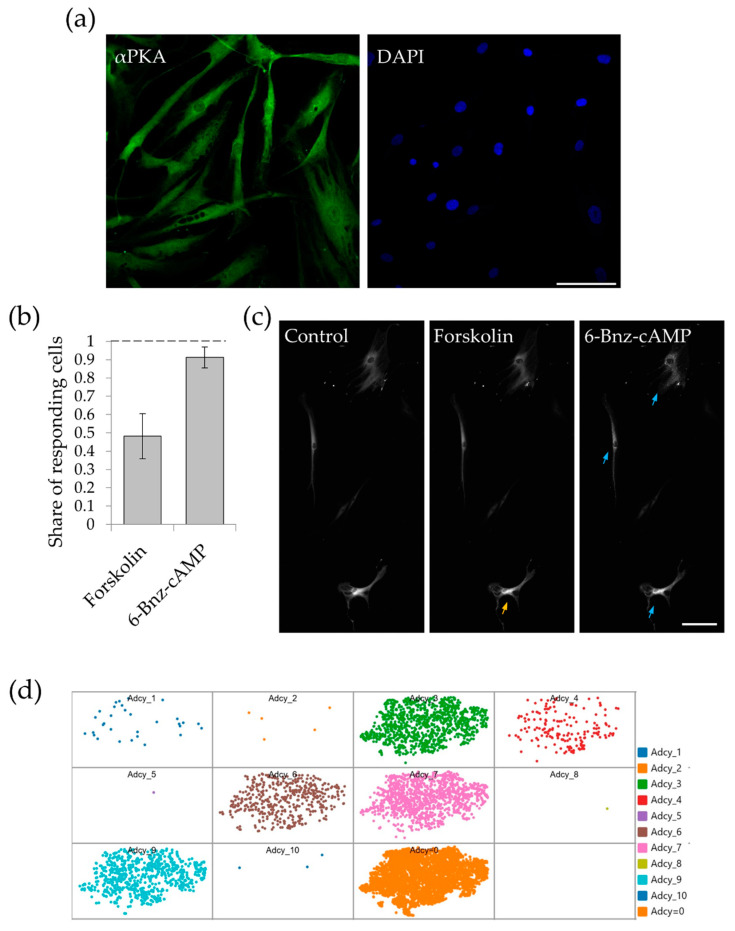
Multipotent stromal cells (MSC) response to adenylate cyclase and protein kinase A (PKA) activation. (**a**) Immunofluorescent staining of the catalytic subunit of protein kinase A in MSC. Scale bar 100 µm. (**b**) Share of the cells responding to adenylate cyclase activator forskolin (10^−6^ M) and cell-permeable direct activator of protein kinase A 6-Bnz-cyclic AMP (10^−4^ M). Results are presented as mean ± SE, *n* = 17–24 in 4 independent experiments on the material of 3 different donors. (**c**) Representative field of view of the cells responding to the sequential adding of forskolin and 6-Bnz-cAMP. Yellow arrow marks the cell responded to the forskolin added. Blue arrows show the cells responded to 6-Bnz-cAMP added. Scale bar 100 µm. (**d**) Expression of mRNA of different isoforms of adenylyl cyclase in human MSC population. Raw data of scRNAseq analysis of MSC were downloaded from Reference [19].

Cultured MSCs uniformly expressed PKA as we demonstrated using immunofluorescent staining of the catalytic subunit of PKA (Figure 1a). Since PKA-Spark biosensor was not used before for analysis of signaling pathways in MSCs, we first examined how cells responded to adenylyl cyclase activator forskolin. Apparently, forskolin induced the formation of SPARK fluorescent aggregates in 48.2 ± 4.7% of cells (Figure 1b,c). These data indicated that almost half of cultured MSCs are not able to activate PKA despite its presence. Direct PKA activation by cell-permeable analogue of cAMP, 6-Bnz-cAMP induced the formation of fluorescent GFP droplets in 91.2 ± 2.8% of MSC (Figure 1b,c), which is consistent with its uniform expression demonstrated by immunofluorescence. Therefore, these data indicate that about half of MSCs lack the activity of adenylyl cyclase. 

Mammalian cells express 10 isoforms of adenylyl cyclase numbered from 1 to 10. To evaluate the expression pattern of adenylyl cyclases in MSC population, we processed the data of single cell RNAseq (scRNAseq) analysis of adipose-derived MSC. The raw data of scRNAseq analysis of both human and mouse adipose-derived MSC are openly available [3,4,19]. Figure 1d shows distribution of human MSC expressing mRNA of different isoforms of adenylyl cyclase in scRNAseq subpopulations. Adenylyl cyclase isoforms from 1 to 10 correspond to *ADCY1* to *ADCY10* genes in humans and *Adcy1* to *Adcy10* genes in mouse. The last subpopulation in the Figure 1d, which is marked as AdCy = 0, shows the cells in which there is no mRNA of all isoforms of these enzymes. The share of MSC lacking the expression of *ADCY1* to *ADCY10* genes in human MSC was 61.0% (5756 from 9429 cells analyzed in dataset from Reference [19]). Distribution of adenylyl cyclases in mouse adipose derived MSC were similar to human one. The share of MSC lacking the expression of *Adcy1 to Adcy10* genes in MSC isolated from mouse subcutaneous adipose tissue were 52.9% (1677 from 3171 cells analyzed in the dataset from Reference [3]) and 75.2% (6151 from 8178 cells analyzed in the dataset from Reference [4]). Thus, more than half of human MSC, as well as mouse MSC, did not express mRNA of any adenylyl cyclase isoforms.

### 2.2. MSC Demonstrate Heterogeneous Response to the Hormonal Stimuli 

**Figure 2:**
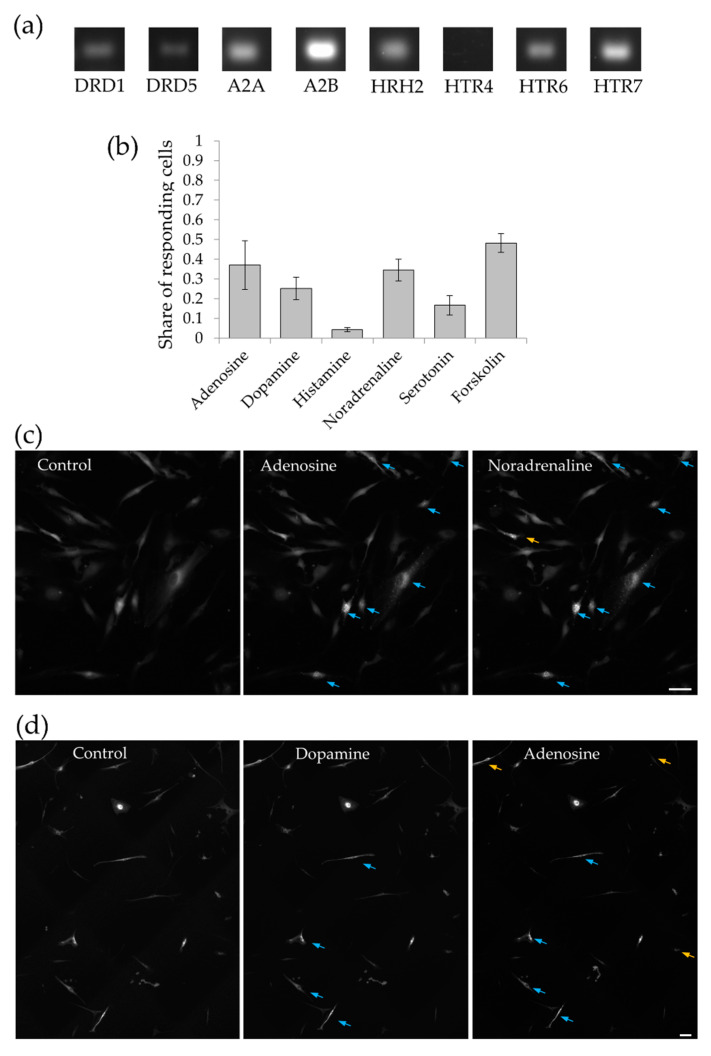
MSC express functionally active receptors activating PKA-dependent signaling. (**a**) PCR analysis of expression of cAMP-dependent receptors to dopamine (DRD1 and DRD5), adenosine (A2A, A2B), histamine (HRH2), and serotonin (HTR6 and HTR7, but not HTR4). (**b**) Share of MSC responding to the hormones with activation of PKA. Mean ± SE, *n* = 4–12 independent experiments on the cells isolated from 3 different donors. (**c,d**) Sequential adding of PKA-activating hormones. The next hormone was added after washing the previous one. Blue arrows mark the cells responded to the first hormone added. Yellow arrows show the cells additionally responded to the second hormone added. Scale bar 100 μm.

Next, we explored PKA activation by the most prominent hormonal MSC activators, including adenosine, dopamine, histamine, noradrenaline, and serotonin, which may activate G_s_-associated isoforms of receptors to these hormones. Expression of G_s_-associated receptors in MSCs was tested using PCR-analysis. Figure 2a, as well as Supplementary Figure S1, shows that MSCs express mRNA of adenosine receptors (A2A and A2B), dopamine receptors (DRD1 and DRD5), histamine receptors (HRH2), and serotonin receptors (HTR6, and HTR7). Among the analyzed receptors, we detected all tested isoforms, with the exception of HTR4 isoform of the serotonin receptor. The expression of all three isoforms of β-adrenoceptors in MSC we recently reported elsewhere [12]. 

MSCs responded to hormones not uniformly, each hormone induced cAMP-dependent formation of GFP aggregates only in the part of cells (Supplementary Figures S2–6, section A in all figures). Evaluating the share of responding cells (Figure 2b), we observed that it does not exceed 40% for any of hormones tested (mean values are 37.0% for adenosine; 34.7% for noradrenaline; 25.2% for dopamine; 16.6% for serotonin; and 4.2% for histamine). The share of the cells responding to adenosine and noradrenaline was close to the number of MSCs responding to forskolin, i.e., which were capable of PKA activation. The close proximity of the numbers of responding cells to the observed maximum may indicate that distinct hormones activate cAMP/PKA pathway in the same cell. To find out whether the same cell responds to different cAMP-activating hormones, we added different hormones sequentially. Due to the biosensor peculiarities, we could not return cells to the basal state by simply washing off added hormones (see Methods section for details). Therefore, we intensively washed the cells before adding the next hormone but did not wait for returning the biosensor to the basal state. As Figure 2c shows, nearly 35% of MSC responded to adenosine, and adding of noradrenaline led to additional activation of only a few percent of the cells. At the same time, a different order of hormonal stimulation, for example, adenosine after dopamine, as shown in Figure 2d, or dopamine after noradrenaline, or adenosine after noradrenaline, and so on, showed the same results: the second hormone activated the same cells as the previous one with only a small addition. We did not see additivity in cellular responses and the overall share of responding cells was consistent with the observation that only about 40% of MSCs are capable of cAMP/PKA activation.

Interestingly, noradrenaline induced fast dissolving of fluorescent droplets in some previously stimulated cells, which means predominantly activation of G_i_-associated α2-adrenoceptors in these particular cells (Supplementary Figure S7). That also means that long-term preservation of the fluorescent droplets after washing the hormone corresponds to the real dynamics of PKA-dependent phosphorylation but is not a biosensor-dependent artifact. 

**Figure 3:**
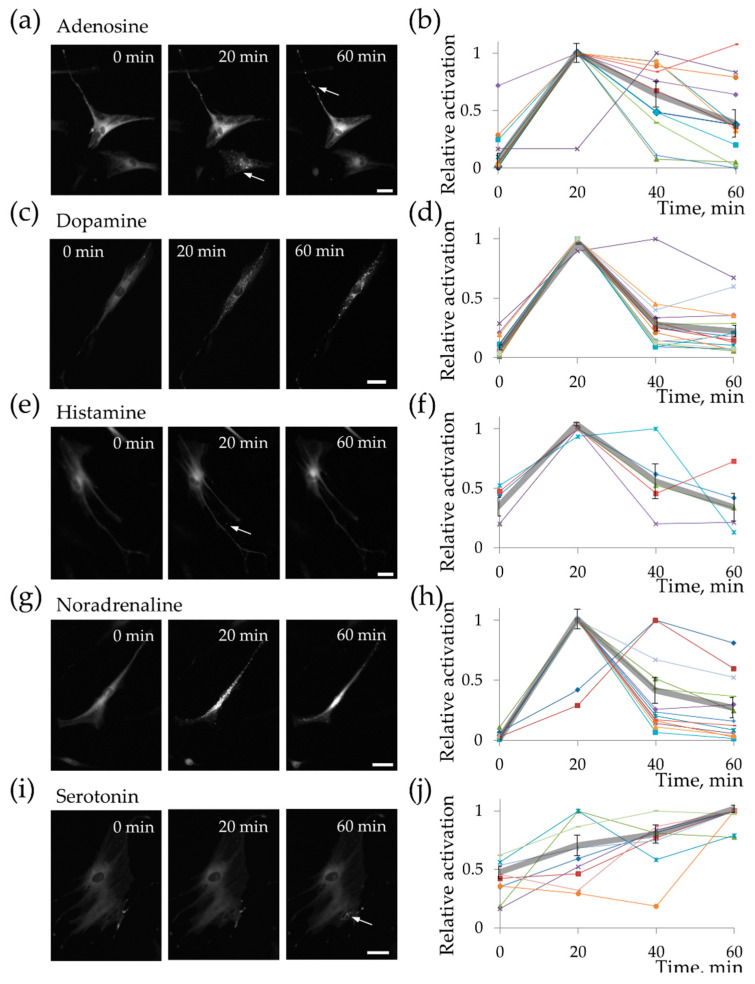
Hormones activated PKA with different amplitude and duration. Responses on (**a**,**b**) 10^−5^ M of adenosine; (**c**,**d**) 10^−5^ M of dopamine; (**e**,**f**) 10^−6^ M of histamine; (**g**,**h**) 10^−6^ M of noradrenaline; (**i**,**j**) 10^−5^ M of serotonin. Left panel shows time points of response of representative cells. Scale bar 20 µm. Right panel shows time dynamics of responses of individual cells (colored thin lines) and corresponding mean response (thick gray line). Mean ± SE, *n* = 5–15 from 2 independent experiments on 2 different donors.

More detailed examination of cAMP-mediated responses of MSC showed some patterns in cellular responses to individual hormones. Adenosine, dopamine, and noradrenaline induced strong and transitory responses, whereas histamine and serotonin activated long but weak cAMP-dependent responses (see Figure 3 and Supplementary Figures S2–S6). Interestingly, some particular cells activated PKA for a long time even in response to adenosine, noradrenaline, and, in one case, dopamine (see Figure 3b,d,h, respectively). Graphs on Supplementary Figures S2–S6 (section (b) in all figures) show the results presented in a different fashion, where it is possible to compare signal amplitudes. It is clear that long responses predominantly have much lower amplitude compared to transitory signals. Thus, MSCs respond to adenosine, dopamine, histamine, noradrenaline, and serotonin, but the amplitude and temporal dynamics of the signal vary between different hormones, as well as between the responses of different cells to a particular hormone. 

### 2.3. MSC Functional Heterogeneity Is Highly Dynamic

**Figure 4:**
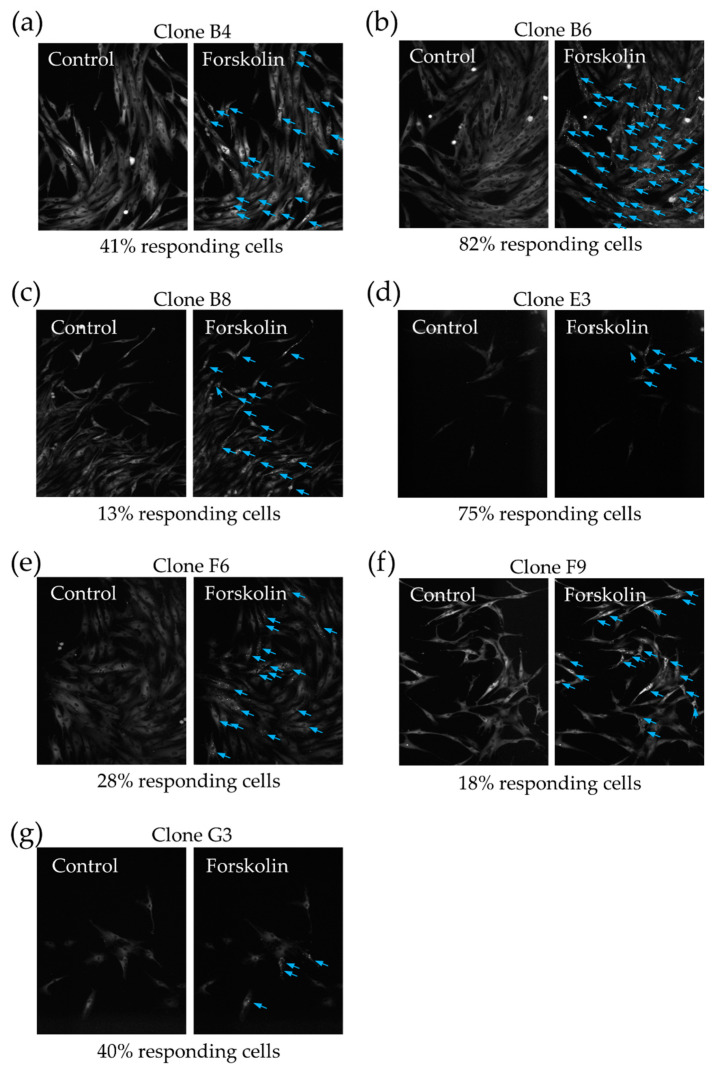
Responses on the forskolin adding to single cell-derived clones of MSC expressing PKA-Spark. Blue arrows mark responding cells. Scale bar 100 µm. (**a**–**g**) representative images of responses of 7 distinct MSC clones. The ratio of responding cells indicated on each panel.

The lack of sensitivity to cAMP-dependent signals in some MSCs can be either a dynamic property of the entire population or stable characteristic of some groups of the cells. To find out whether MSC are able to form functional heterogeneity with respect to cAMP-dependent signaling, we obtained single cell-derived clones of MSC expressing PKA-Spark. We used immortalized adipose-derived MSC to get higher efficiency of clonal growth. Cells were analyzed two weeks after sorting 1 cell per well. The entire plate with grown MSC clones is shown in Supplementary Figure S8. The cells from one clone showed similar phenotype regarding size, morphology, and PKA-Spark expression level (see Figure 4). At the same time, forskolin stimulation led to PKA-Spark response in 43.6 ± 7.5% of the cells of clones, which is highly consistent with the data obtained in the total population. Thus, functional heterogeneity of MSC seems to be acquired during cooperative growth of the cells, even in the case of genetically identical cells from one clone.

## 3. Discussion

MSCs carry out a huge number of functions in the tissues they locate, from differentiation into several mesenchymal directions to regulation of a number of physiological functions, such as angiogenesis, neurogenesis, modulation of immune response, and regulation of tissue specific stem cells differentiation. It is hard to imagine that every cell of the MSC population performs all these functions. An idea of heterogeneity of the MSC population is rather old, but attempts to link the particular cell’ phenotype with its function or sorting of the MSCs to functionally homogeneous sub-populations were often without effect. Several attempts, however, were successful. Mendez-Ferrer and colleagues described nestin-positive subpopulation of bone marrow derived MSC, which uniquely transmits regulatory signals from sympathetic nervous system to hematopoietic stem cells [17]. Recently, using the separation of the MSC population based on the hormonal sensitivity of individual cells and their signaling properties, we identified a subpopulation of MSC, which differentiates into adipocytes faster and more effectively than other MSC, but, at the same time, they are not committed preadipocytes [7]. These cells differ from other MSC by co-expression of angiotensin II receptors of types 1 and 2, which can heterodimerize and change signaling properties of MSC and their response to differentiation stimuli [20]. Responsivity to the hormonal stimuli and the type of response define functional activity of MSC; thus, it is a sensitive and effective approach to study functional heterogeneity of MSC. cAMP-dependent signaling is activated by a number of critical regulators of MSC functional activity, such as noradrenaline or adenosine. Moreover, as we recently showed, activation of cAMP/PKA-dependent signaling leads to heterologous sensitization of MSC and potentiation of α1A-adrenoceptor/Ca^2+^-dependent response [15].

In this work, we demonstrated, for the first time, heterogeneity of hormone-activated cAMP-dependent responses of MSC at the single cell level. We showed that MSC responded to the hormones heterogeneously, and different hormones activated PKA with different time dynamics and amplitude. Moreover, individual cells may respond to the same hormone differently. This variable signaling may play a crucial role in the ability of cells to react to the complex hormonal regulation in the tissue. cAMP-dependent signaling lacks the unparalleled specificity and networking of signaling pathways associated with receptor Tyr-kinases, as cAMP elevation mainly affects PKA. However, PKA specificity may be regulated by temporal profile of PKA activation and through its interaction with A-kinase anchoring proteins (AKAPs), which is mediated by diversity of AKAP isoforms [21]. Thus, responsive cells are capable of sensing more than one hormone at the time and forming a wide variety of cellular answers, depending on external hormonal stimuli. These findings on type and format of cAMP-dependent response of particular cells may be associated with the functional activity of tissue, but this requires further research.

Signaling heterogeneity of PKA-dependent response is due to the lack of adenylyl cyclase in some MSCs. This is an extremely unexpected result indicating a possible new level of the regulation of cAMP/PKA signaling by controlling the availability of adenylyl cyclase. Direct analysis of adenylyl cyclase expression at the single cell level is complicated by the abundance of adenylyl cyclase isoforms in mammalian cells [22]. Although methods of multiplex protein analysis at the single cell level have already appeared, these approaches are still laborious and low-accessible. Another possible approach to the analysis of protein expression in both multiplex and single cell modes, is single cell RNAseq. To partially testify our findings about heterologous activity of adenylyl cyclases, we analyzed openly available results of single cell RNAseq of human and mouse adipose derived MSCs [3,4,19]. We found near 60% of the cells that lacked mRNA of all isoforms of adenylyl cyclase (*Adcy1* to *Adcy10* genes in mice and *ADCY1* to *ADCY10* genes in humans). Although mRNA level does not necessary reflects the expression of corresponding protein, especially in case of their detection at the single cell level, we suggest that cells lacking adenylyl cyclase mRNA may have a reduced level of these proteins; thus, they do not respond to cAMP/PKA-mediated hormonal stimuli. 

There were some reports about heterogeneous expression and responses of adenylyl cyclases in clonally derived cells [23]. Due to the lack of reliable probe for registration of cAMP production or PKA activity at single cell level, the authors obtained single cell derived clones to compare cell-to-cell heterogeneity indirectly. Here, we showed that PKA-mediated responses are varied even between the cells of one clone. PKA-associated signal heterogeneity can be formed during the proliferation of single-cell derived clones, which means that this heterogeneity is a dynamic process. Thus, it can indicate that MSCs are capable of self-organization and specialization via cell-to-cell interactions (probably both contact and paracrine). This phenomenon may determine the ability of MSC to form cell niches in tissues where cells can form a complex system composed of cells primarily attributing to the reception of stimuli and cells dedicated to driving tissue cell renewal via proliferation and differentiation.

PKA signaling heterogeneity forming in cell clones can be viewed as a manifestation of «gene expression noise» [24]. Previously, a similar phenomenon was described in regard to stem cell marker Sca-1 [25]. It is known that progenitor cells demonstrate stochasticity in gene expression, which results from fluctuations in transcription and translation [26]. Moreover, this stochasticity plays an important role in maintaining the multipotency of stem cells and decreases as cells become more and more lineage-restricted [27].

## 4. Materials and Methods 

### 4.1. MSC Culture

Primary cell line of MSC isolated from adipose tissue of healthy donors was obtained from the biobank of the Institute for Regenerative Medicine, Medical Research and Education Center, Lomonosov MSU, collection ID: MSU_MSC_AD. All procedures performed with tissue samples from patients were in accordance with the Declaration of Helsinki and approved by the Ethic Committee of Medical Research and Education Center, Lomonosov Moscow State University (IRB00010587), protocol #4 (date of approval 4 June 2018), and all donors provided informed consent. Telomerase expressing immortalized MSC (hTERT-MSC) were obtained from ATCC (ATCC ASC52telo). 

Cells were cultivated in AdvanceSTEM medium (HyClone, Logan, UT, USA) supplemented with 10% Mesenchymal Stem Cell Growth Supplement (HyClone, Logan, UT, USA), 1% Penicillin/Streptomycin solution (Gibco, Logan, UT, USA), 1% L-glutamine (Gibco, Logan, UT, USA) under an atmosphere of 5% CO_2_ at 37 °C. Culture medium was renewed every 2 to 3 days. Cells were passaged every 4 days, with a subcultivation ratio of 1:3.

### 4.2. RNA Isolation and PCR Detection of Receptors

The expression of receptors coupled with intracellular PKA-signaling was evaluated using RT-PCR. Total RNA was isolated from cells using RNeasy Mini Kit (Qiagen, Germantown, MD, USA) according to the user manual. 1 µg of total RNA was used for reverse transcription using MMLV Reverse transcription kit (Evrogen, Moscow, Russia). Resulting cDNA was used as a template for PCR with specific primers pairs (Evrogen, Moscow, Russia; see Table 1 for details). All specified procedures were carried out according to the manufacturer’s instructions. PCR products were then analyzed by electrophoresis in the agarose gel (2% agarose, visualization with EtBr), and product length was estimated using GeneRuler 50 bp DNA Ladder (Thermo Fisher, Logan, UT, USA).

### 4.3. Lentiviral Transduction of MSC

To deliver the cAMP sensor to MSCs, it was cloned from pcDNA3-PKA-SPARK (#106920, Addgene, Watertown, MA, USA) into the LeGO-iG2 lentiviral transfer vector (#27341, Addgene, Watertown, MA, USA) using restriction endonucleases MluI and EcoRI. Lentiviral particles (LVPs) were assembled in HEK293T cells using the standard PEI transfection protocol [28]. The conditioned medium containing LVPs was collected 48–72 h post-transfection and separated from cell debris by centrifugation at 4000× *g* 4 °C for 15 min. In order to increase the efficiency of transduction, protamine sulfate (50 µg/mL) was added to the medium containing LVPs. An immortalized MSC line (ASC52telo) or MSC of early (<3) passages were used for transduction. Cells cultured on 24-well plates were incubated in the medium containing LVPs and subjected to centrifugation at 800× *g* RT for 1.5 h to assist virus entry. After centrifugation, the medium was changed to standard culture medium and cells were incubated for ~8 h. The transduction procedure was repeated 2 times. Between transductions, the virus stock was stored at +4 °C. 

### 4.4. Registration of PKA Activation with PKA-Spark

To registrate PKA activation with PKA-Spark, we grew transduced cells in 24- or 48-well plates at low densities to prevent cell-to-cell communications during imaging of signaling. Before the experiment the growth medium was changed to Hanks Balanced Salt Solution (PanEco, Moscow, Russia) with 20 mM Hepes (HyClone, Logan, UT, USA). To analyze the functional activity, cells were treated with 10^−5^ M of adenosine (Abcam, Cambridge, UK), 10^−5^ M of dopamine (Tocris Bioscience, R&D Systems, Minneapolis, MN, USA), 10^−6^ M of histamine (Abcam, Cambridge, UK), 10^−6^ M of noradrenaline (Abcam, Cambridge, UK), 10^−5^ M of serotonin (Abcam, Cambridge, UK), 10^−6^ M of forskolin (Abcam, Cambridge, UK), or 10^−4^ M of direct activator of protein kinase A 6-Bnz-cAMP (Biolog, Bremen, Germany) after registration of basal activity of the cells. Activation of PKA was measured in individual cells using an inverted fluorescent microscope Nikon Eclipse Ti equipped with an objective CFI Plan Fluor DLL 10X/0.3 (Nikon, Tokyo, Japan) and with digital cooled monochrome CCD camera Nikon DS-Qi1 (Nikon, Tokyo, Japan). We used the simultaneous measuring of 6 × 6 fields of view in Large Image mode to increase the number of analyzed cells. Movies were analyzed using NIS-Elements (Nikon) and ImageJ software (NIH, Bethesda, MD, USA).

**Figure 5:**
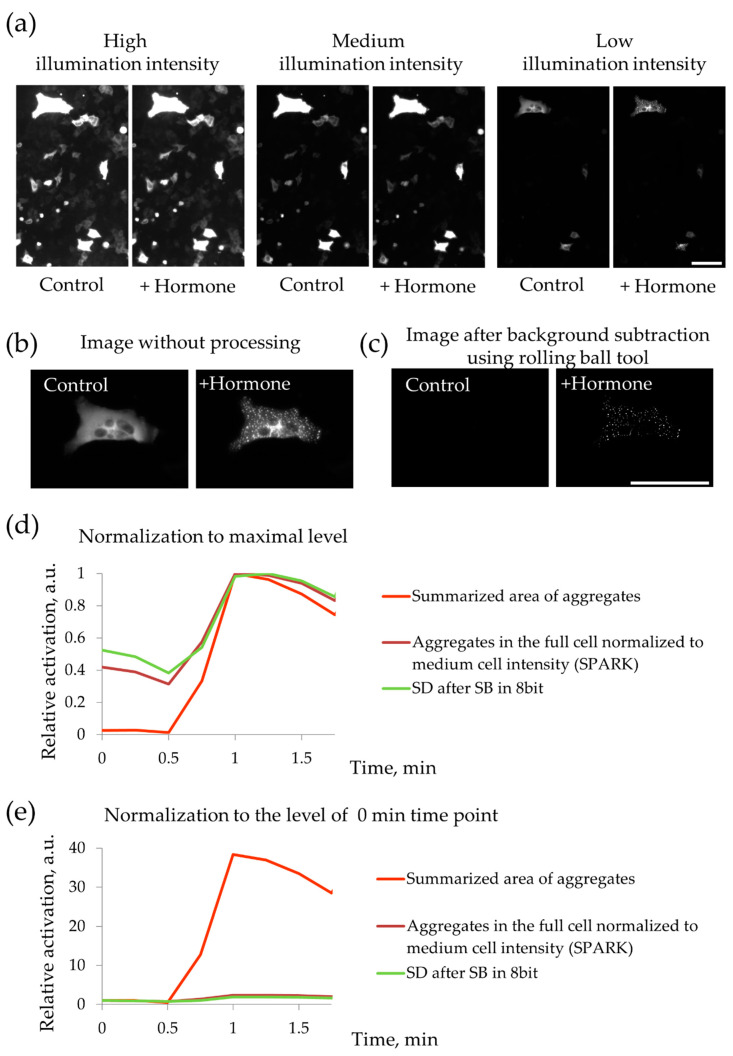
Testing the method of registration of PKA activity using PKA-Spark fluorescent probe. (**a**) PKA-Spark was expressed in HeLa-Kyoto cells with different expression levels and the cells were stimulated with serotonin (10^−5^ M). Fluorescence of the same field of view was registered with different illumination intensity (marked above the pictures). It is seen that only the most bright cells expressing the probe with relatively high level, demonstrated hormone-dependent response. Scale bar 100 µm. (**b**,**c**). Processing of fluorescent images of responding cells. (**b**) Responding cell before and after hormone adding without image processing. (**c**) Fluorescent figures after background subtraction using rolling-ball tool. Scale bar 100 µm. (**d**,**e**) Test of different strategies of quantification PKA-Spark fluorescent droplets formation. (**d**) The results of quantitative analysis were normalized to the maximum measured level. (**e**) The results of quantitative analysis were normalized to the zero time point.

To optimize the detection of local PKA activation using PKA-Spark biosensor, we used the HeLa-Kyoto cell line. These cells were transfected with PKA-Spark encoding plasmid using FuGENE HD transfection reagent (Promega, Logan, UT, USA) according to manufacturer’s instruction. Twenty-four hours after transfection, we stimulated these cells by serotonin and registered the formation of fluorescent aggregates in the cytoplasm, which corresponded to the activation of G_s_/AC/PKA-dependent signaling. Importantly, only cells with high PKA-Spark expression level demonstrated the formation of aggregates in response to the hormone (Figure 5). The response of other cells was possible to register only 1–2 days later, when PKA-Spark expression increased sufficiently. Therefore, only cells with enough level of biosensor expression should be analyzed. For further analysis of MSC responses, we first identified the threshold level of biosensor expression, which allows the detection of the formation of fluorescent aggregates in the cytoplasm, and only cells with PKA-Spark expression exceeding established threshold were taken into analysis. To optimize the quantification of fluorescent aggregates formation, we compared 3 alternative approaches: 1—SPARK signal was quantified as the sum of fluorescent droplets’ pixel intensity divided by sum of cells’ pixel intensity [18]; 2—signal was quantified as changes in standard deviation (SD) of the picture intensity; and 3—SPARK signal was calculated as the sum of the area of all fluorescent droplets after background subtraction using a “rolling ball” tool (ImageJ software, Bethesda, MD, USA) (Figure 5b–e). We found out that the latter approach, a calculation of total area of fluorescent aggregates, shows the minimal level of signal in the absence of stimulation (Figure 5d) and the highest contrast (Figure 5e). Therefore, for further analysis, we used this approach to quantify MSC responses. Images were processed using ImageJ software. We also analyzed the signal reversibility and found that PKA-Spark demonstrates a very slow dynamics of the fluorescent droplets disassembly, and the droplets partially remain 30 min after washing off the hormone. Thus, the repetitive hormonal stimulation of the same cells which is commonly used during calcium signaling registration [10] is not possible for this detection method. 

### 4.5. Immunofluorescent Analysis of PKA Expression in MSCs

Cells were fixed in 4% paraformaldehyde for 10 min. After several washes by phosphate buffer saline (PBS) (PanEco, Moscow, Russia), cells were incubated in 0.1% bovine serum albumin (BSA) containing 10% normal donkey serum to block non-specific binding of antibodies. This was followed by incubation with specific primary antibodies against PKA (PA5-70360, Thermo Fisher Scientific, Logan, UT, dilution 1:100), for 1 h and subsequent extensive washing in PBS. Then, cells were incubated with Alexa488-conjugated donkey anti-rabbit (Molecular Probes, Logan, UT, USA). Cell nuclei were counterstained with DAPI (Sigma-Aldrich, St. Louis, MO, USA) and mounted in Aqua Poly/Mount (Polysciences Inc, Warrington, PA, USA). For negative controls, rabbit non-specific IgGs were used in appropriate concentration. Images were obtained using the confocal microscope LSM 780 and ZEN2010 software (Zeiss, Oberkochen, Germany).

### 4.6. scRNAseq Data Processing

To analyze patterns of different isoforms of adenylyl cyclases expression at the single cell level, we used freely available data of single cell RNAseq analysis of adipose-derived MSC. Raw data were downloaded from databases ArrayExpress (EBI) и GEO (NCBI, SRA): mouse-derived MSC (E-MTAB-6677) [3], (SRP145475) [4], and human-derived MSC (SRP148833) [19]. Downloaded datasets were processed using Cellranger 3.0 software with standard parameters, and analysis of cloupe files was performed using Loop Browser 4.0 software (10x Genomics, San Francisco, CA, USA). The number of the cells lack of adenylyl cyclases mRNA was counted using ‘Filter’ tool in Loop Browser 4.0 and filtering on adenylyl cyclase isoforms genes *Adcy1* (*ADCY1* in case of human MSC population analysis) to *Adcy10* (*ADCY10* in case of human MSC).

### 4.7. MSC Sorting and Clonal Analysis 

To obtain single cell derived clones of PKA-Spark expressing cells, hTERT-MSC 6 days after lentiviral transduction were re-suspended in BSA buffer (DPBS, 0.1% BSA) and the 10% of the brightest cells were sorted in single-cell mode separately into 96-well tissue culture plates. Cells were sorted based on fluorescence in 488-530/30 channel on a BD FACSAria III sorter. Doublets were excluded based on side scatter height and width. Then, single clones were cultured in AdvanceSTEM full growth medium for two weeks. The medium was changed to the fresh every 3–4 days.

### 4.8. Statistics

The share of responding cells was calculated as the ratio of the number of cells that formed fluorescent droplets in response to the hormonal stimulation, to the number of all cells in which brightness exceeds the established threshold level of biosensor expression.

## 5. Conclusions

This study demonstrates that cAMP-activating hormones could exert their cAMP-dependent effects only in about half of MSCs. At the same time if a particular MSC activates cAMP production in response to one cAMP-activating hormone, it would respond to another one as well. Surprisingly, the heterogeneity of hormone-activated cAMP-dependent responses results from various availability of adenylyl cyclases. Thus, our data indicate that despite generally accepted redundancy and ubiquitous expression of adenylyl cyclase isoforms, the presence of this enzyme in particular MSC determines their ability to respond to hormones by activation of PKA/cAMP signaling. Interestingly, this signaling heterogeneity observed in total population re-establishes in single-cell derived clones. This may underline heterogeneous functional responses of MSC to hormones. Molecular mechanisms of establishing of MSC heterogeneity caused by the regulation of adenylyl cyclase expression as well as functional consequence of such heterogeneity need to be further elucidated. 

## Figures and Tables

**Table 1:** Primers used for receptors expression analysis.

Target	Forward Primer	Reverse Primer	T Annealing	Product Length
HRH2	CGT GTC CTT GGC TAT CAC TGA	GGC TGG TGT AGA TAT TGC AGA AG	55 °C	119 bp
HRH2	CAG TTC GGG TCG CCA TCT C	CTG GTC TCG TTC CTG CTG TTC	55.2 °C	100 bp
DRD1	AGG GAC TTC TCT GTT CGT ATC C	AGG GAC TTC TCT GTT CGT ATC C	54.9 °C	103 bp
DRD1	TCT GTG CTG CCG TTA TCA GG	TTG TGG GTT TTG CCT TGT GC	54.2 °C	378 bp
DRD5	CCG TGT CAG ACC TTT TCG TG	TGC GCT GAG TCA TCT TGC G	54.7 °C	216 bp
DRD5	GGG CAG TTC GCT CTA TAC CAG	GGT CCA GAT GAT GAG TAG GGT C	56 °C	126 bp
A2B	CTG TCA CAT GCC AAT TCA GTT G	GCC TGA CCA TTC CCA CTC TTG	55 °C	134 bp
A2A	CGC TCC GGT ACA ATG GCT T	TTG TTC CAA CCT AGC ATG GGA	54.5 °C	109 bp
A2A	CTG GCT GCC CCT ACA CAT C	TCA CAA CCG AAT TGG TGT GGG	54.3 °C	116 bp
HTR4	CTC ACG TTT CTC TCG ACG GTT	AGC AGA TCC GCA AAA GCA AGA	54.6 °C	137 bp
HTR4	GAT CTG CTG GTT TCG GTG CT	CAG AGG GGT CAT CTT GTT CCT A	54.6 °C	213 bp
HTR6	GCA ACA CGT CCA ACT TCT TCC	TGC AGC ACA TCA CGT CGA A	54.1 °C	159 bp
HTR7	CGA AGA TGA TTC TCT CCG TCT G	GCG GTA GAG TAA ATC GTA TAG CC	54.9 °C	136 bp
HTR7	TGG TGA TCT CCG TGT GCT TC	TCC AAA GAT CCA CTT GCC CC	54.8 °C	149 bp

## Supplementary Materials

Supplementary Materials can be found at https://www.mdpi.com/1422-0067/21/12/4442/s1.

## Abbreviations

MSC	Multipotent stromal cells, Mesenchymal stem/stromal cells
PKA	Protein kinase A
AC	Adenylyl cyclase
