# Supplementary material for: Functional Heterogeneity of Protein Kinase A Activation in Multipotent Stromal Cells
Source: Int J Mol Sci. 2020 Jun 22;21(12):4442. doi: 10.3390/ijms21124442 (PMC7353043; doi:10.3390/ijms21124442)
Supplement: Supplementary file 1 [file ijms-21-04442-s001.pdf]

(a)

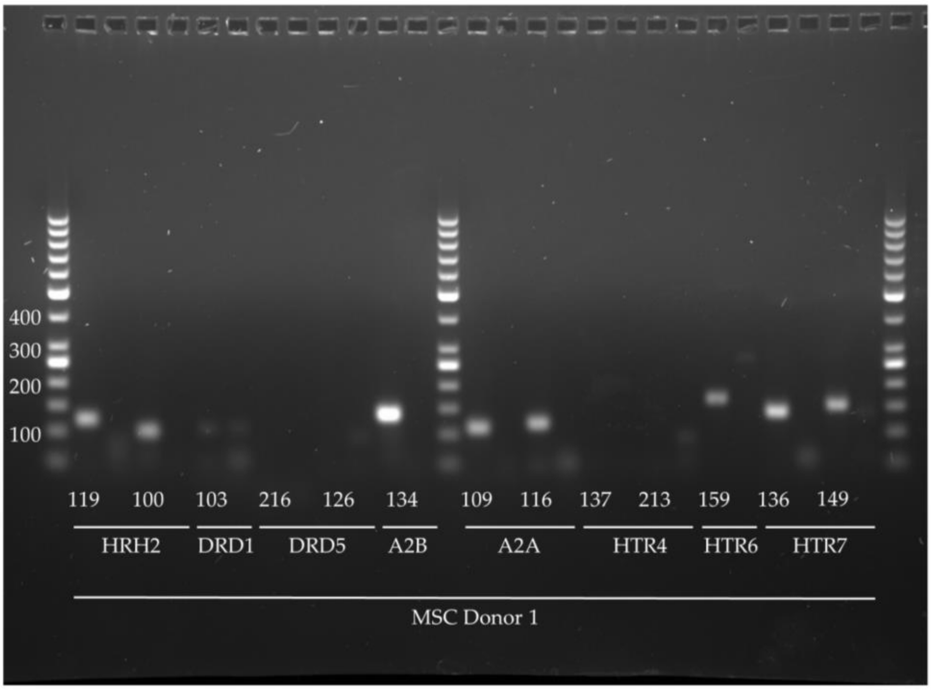

(b)

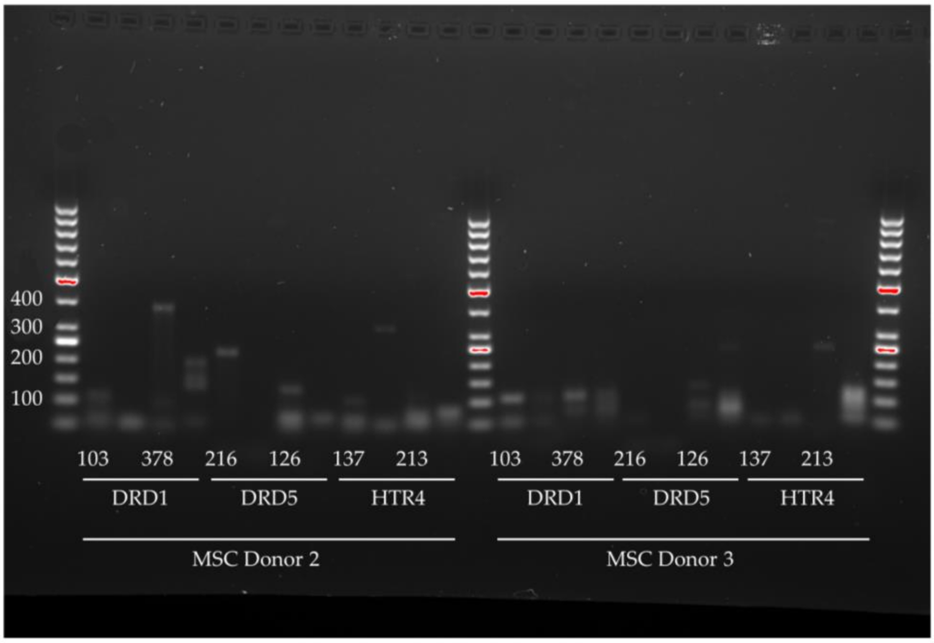

Supplementary Figure S1. Full gels of PCR analysis of expression of cAMP-dependent receptors to dopamine (DRD1 and DRD5), adenosine (A2A, A2B), histamine (HRH2), and serotonin (HTR6 and HTR7, but not HTR4). (**a**) The first gel. (**b**). The second gel. 'MSC donor 1', 'MSC donor 2', and 'MSC donor 3' correspond to MSC populations isolated from three different donors. The numbers marked under the gel tracks correspond to the products molecular weights. Molecular weight of gene ruler bands marked to the left of the gels.

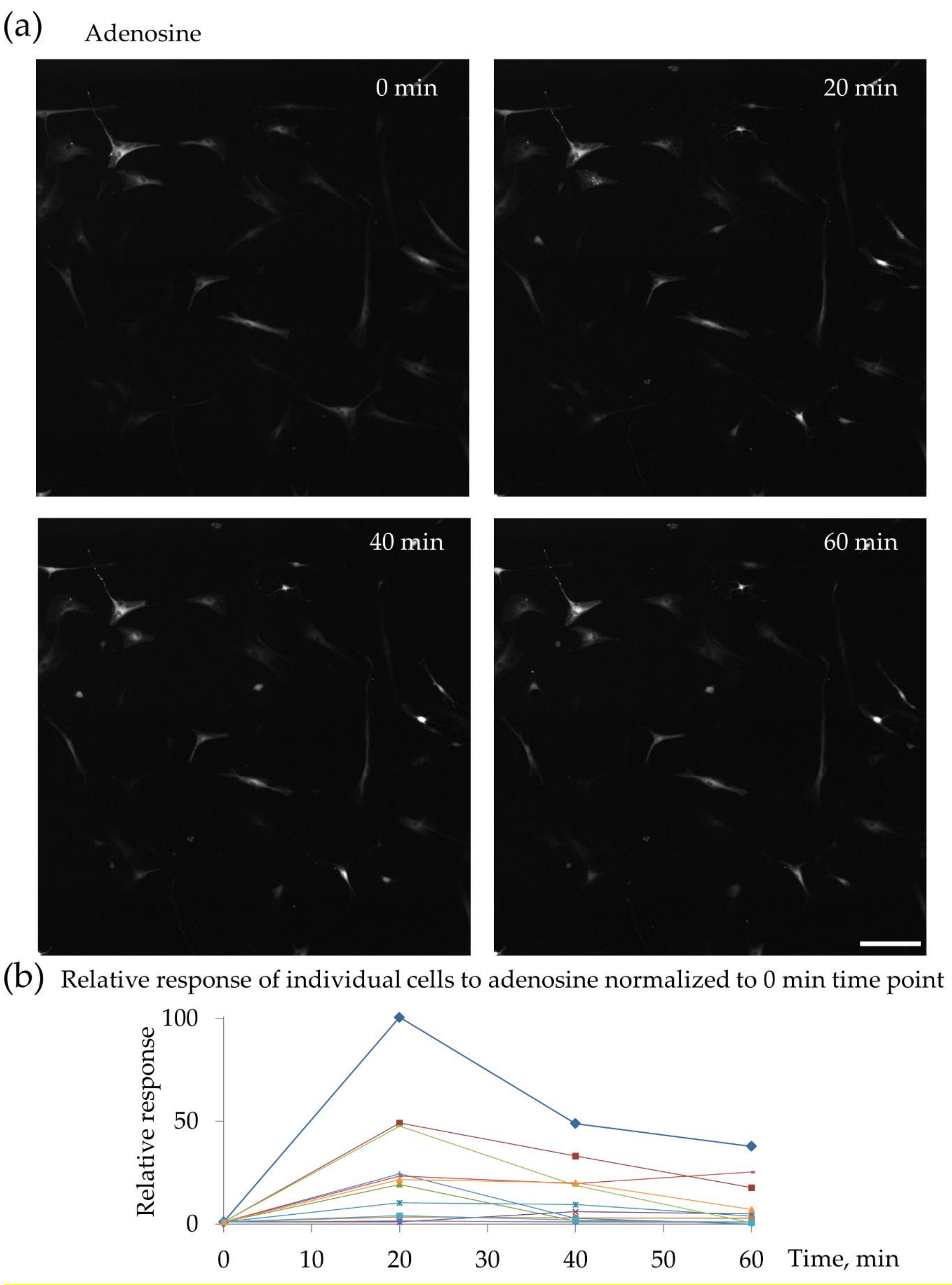

Supplementary Figure S2. Hormones activated PKA with different amplitude and duration. (**a**) Large field of view of the cells stimulated with 10^{-5} M of adenosine. The time after adding the hormone is marked in the upper right corner. Scale bar 100 μm. (**b**) The relative response of individual cells to adenosine, normalized at time point 0 min. The graphs show time dynamics of responses of individual cells (colored thin lines).

(a)

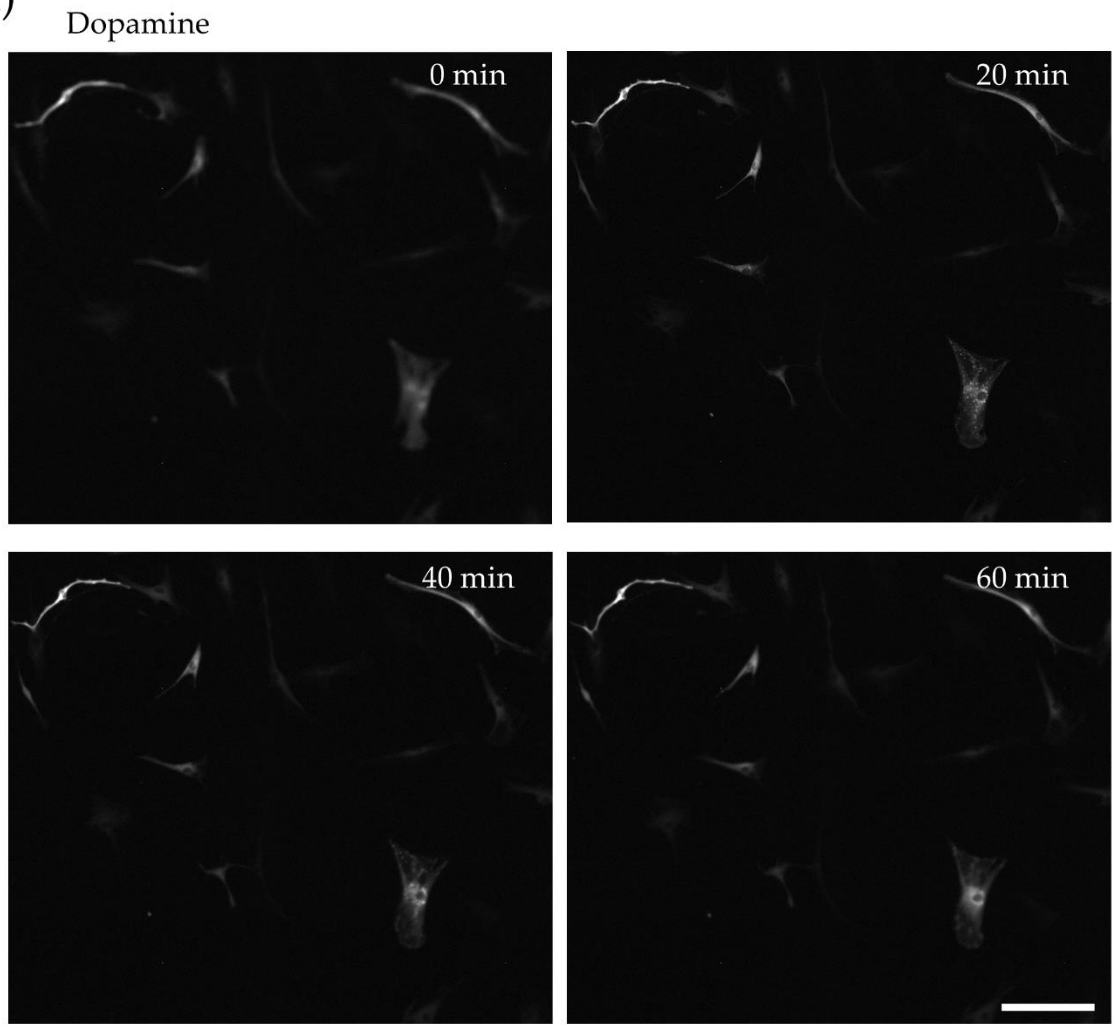

(b) Relative response of individual cells to dopamine normalized to 0 min time point

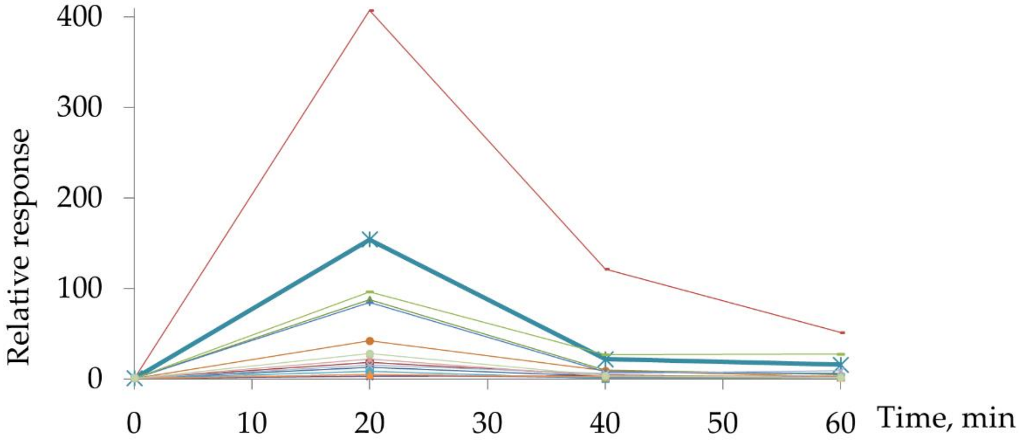

Supplementary Figure S3. Hormones activated PKA with different amplitude and duration. (**a**) Large field of view of the cells stimulated with 10^{-5} M of dopamine. The time after adding the hormone is marked in the upper right corner. Scale bar 100 μm. (**b**) The relative response of individual cells to adenosine, normalized at time point 0 min. The graphs show time dynamics of responses of individual cells (colored thin lines).

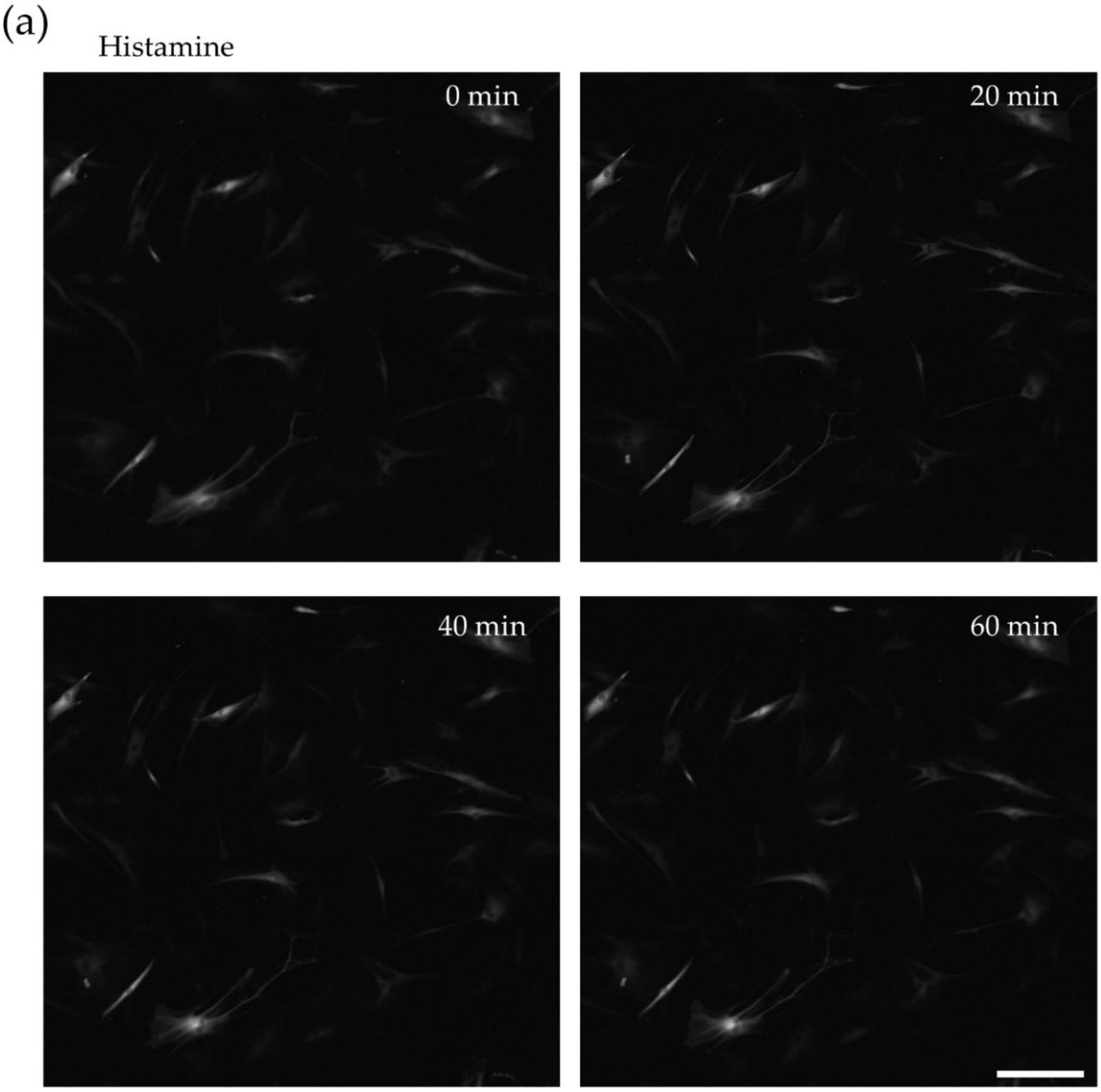

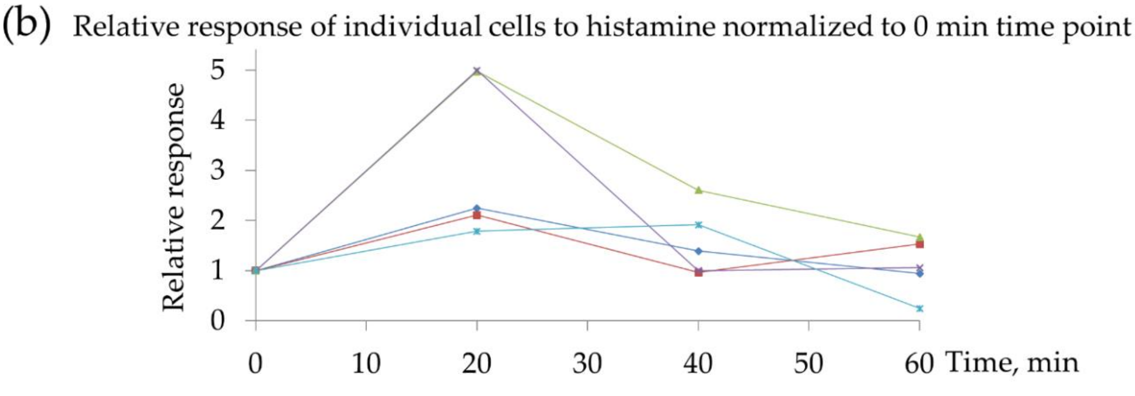

Supplementary Figure S4. Hormones activated PKA with different amplitude and duration. (**a**) Large field of view of the cells stimulated with 10^{-6} M of histamine. The time after adding the hormone is marked in the upper right corner. Scale bar 100 μm. (**b**) The relative response of individual cells to adenosine, normalized at time point 0 min. The graphs show time dynamics of responses of individual cells (colored thin lines).

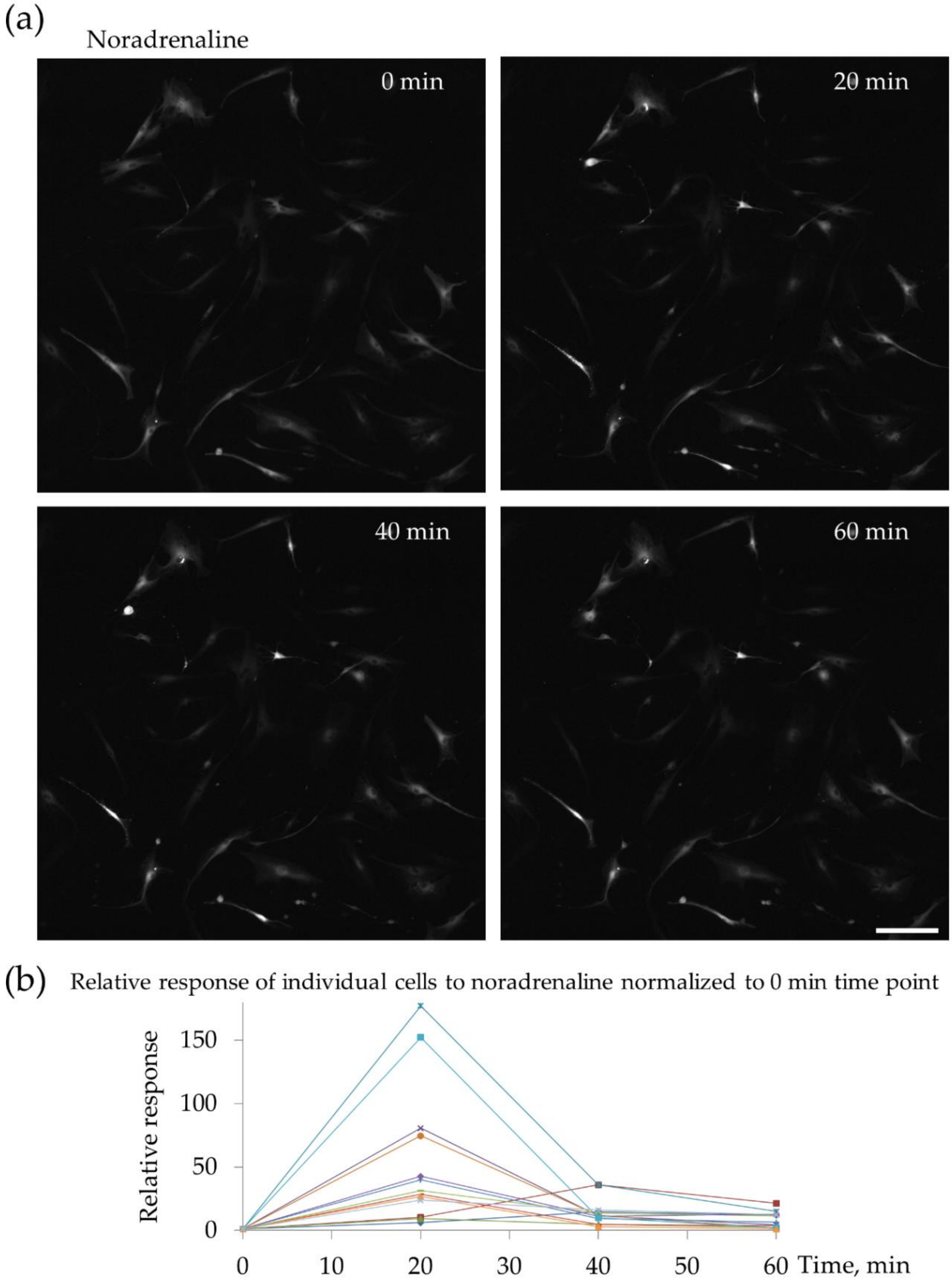

Supplementary Figure S5. Hormones activated PKA with different amplitude and duration. (**a**) Large field of view of the cells stimulated with 10^{-6} M of noradrenaline. The time after adding the hormone is marked in the upper right corner. Scale bar 100 μm. (**b**) The relative response of individual cells to adenosine, normalized at time point 0 min. The graphs show time dynamics of responses of individual cells (colored thin lines).

(a) Serotonin

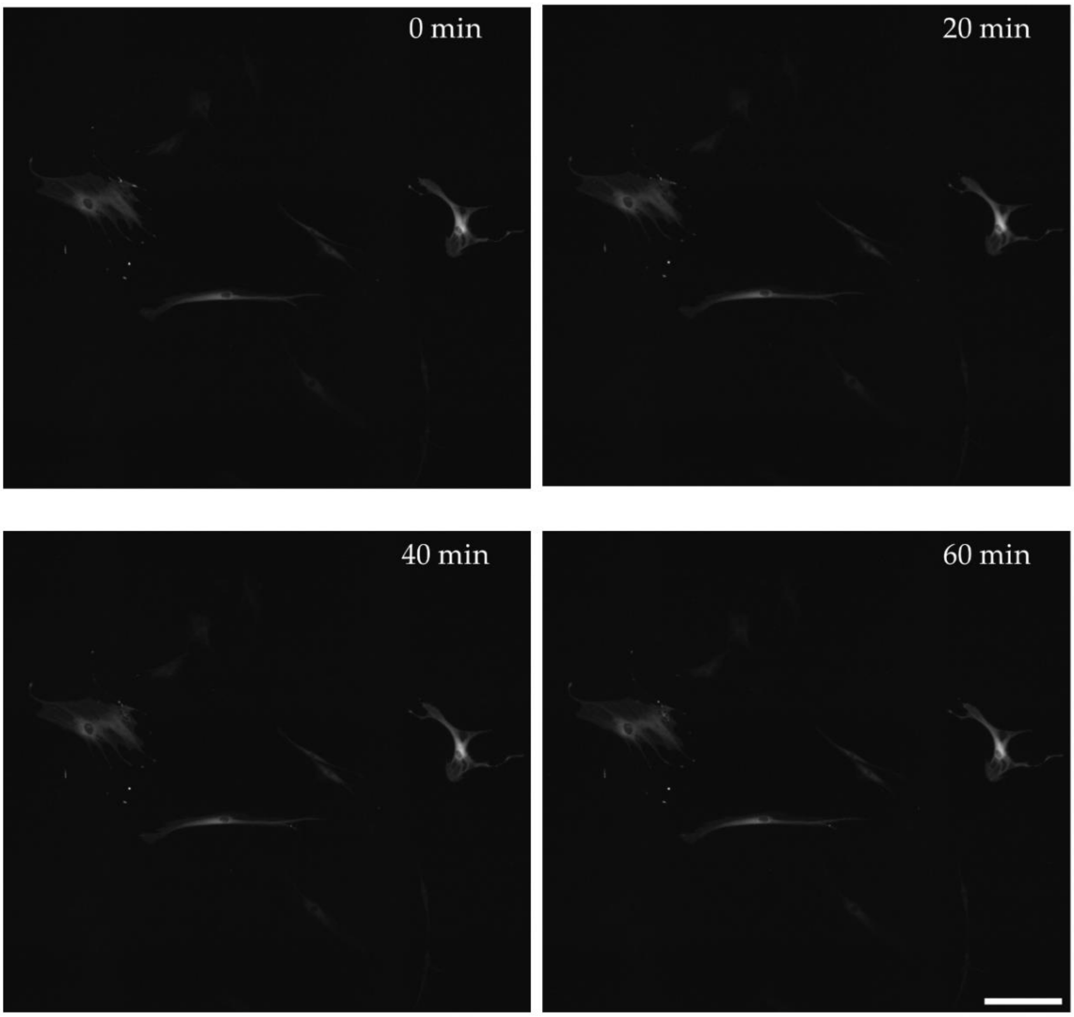

(b) Relative response of individual cells to serotonin normalized to 0 min time point

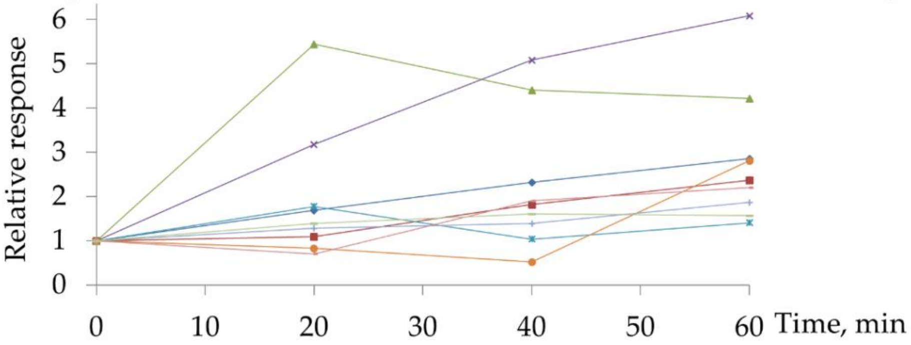

Supplementary Figure S6. Hormones activated PKA with different amplitude and duration. (**a**) Large field of view of the cells stimulated with 10^{-5} M of serotonin. The time after adding the hormone is marked in the upper right corner. Scale bar 100 μm. (**b**) The relative response of individual cells to adenosine, normalized at time point 0 min. The graphs show time dynamics of responses of individual cells (colored thin lines).

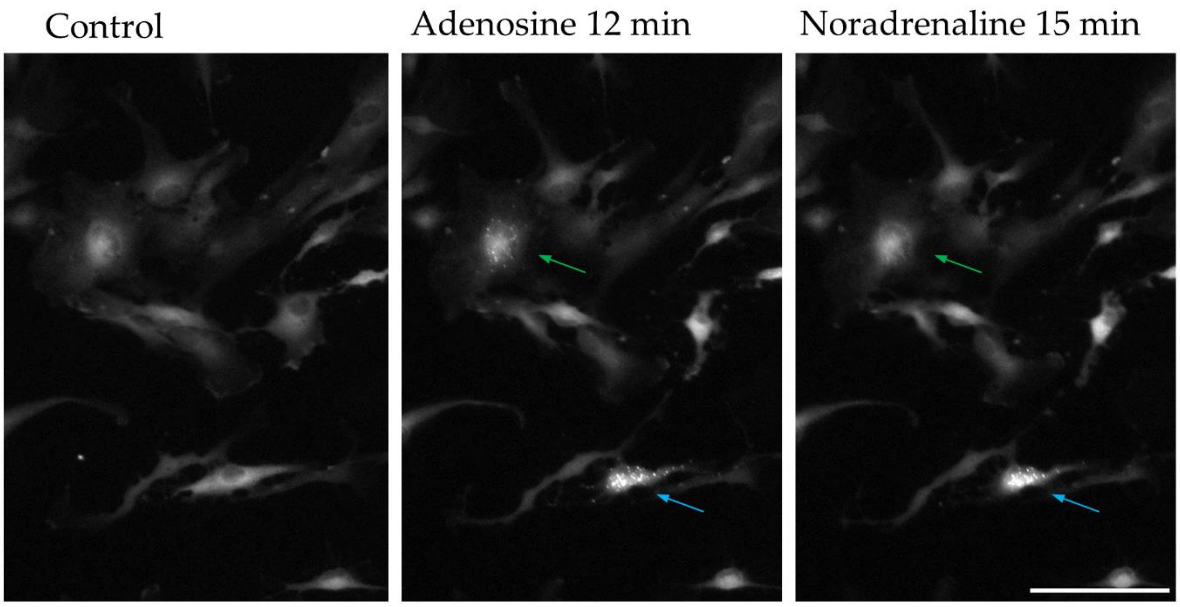

Supplementary Figure S7. Sequential adding of PKA-activating hormones 10^{-5} M of adenosine and then 10^{-6} M of noradrenaline. The next hormone was added after washing the previous one. Blue arrow marks the cell that responded to the first hormone added and remained activated after the addition of noradrenaline. Yellow arrow shows the cell in which fluorescent droplets dissolved after the addition of noradrenaline. Scale bar 100 μm.

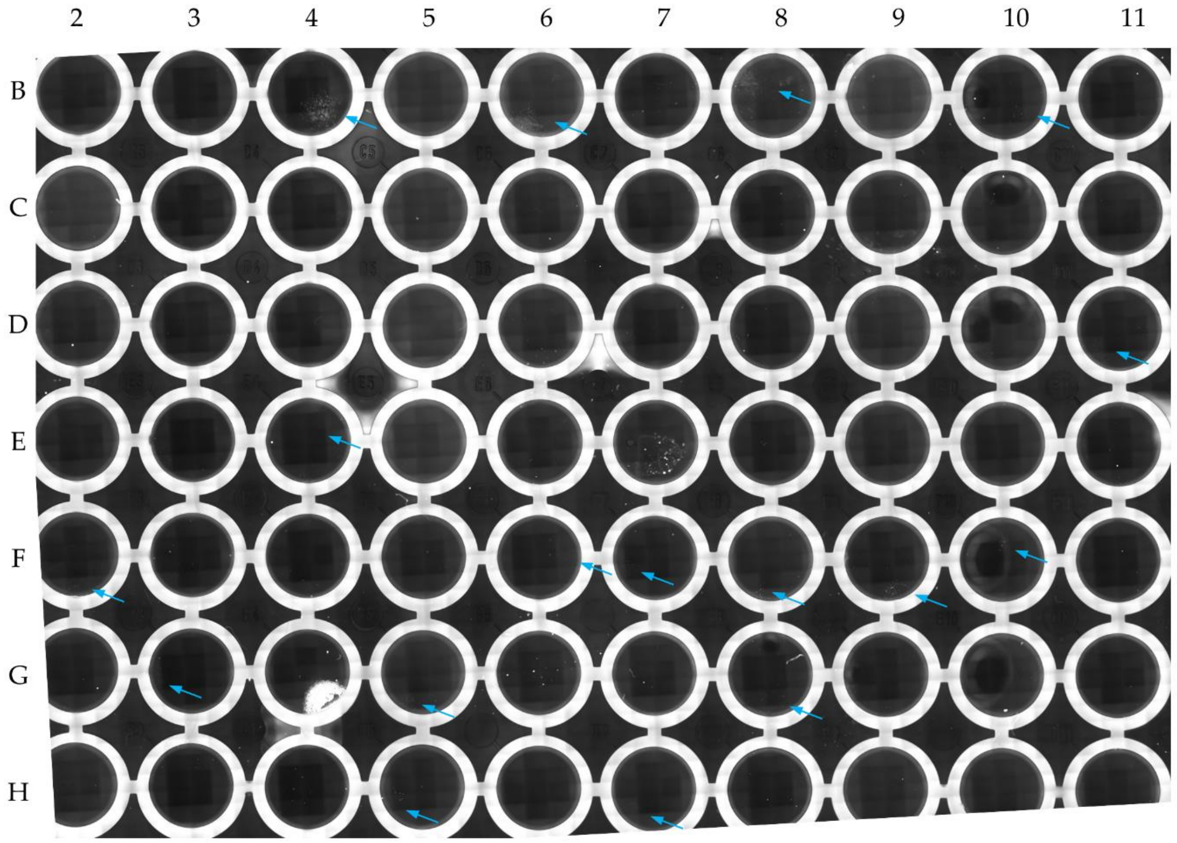

Supplementary Figure S8. The entire plate with grown MSC clones. Blue arrows indicate the wells containing MSC clones. The name of a particular clone consists of the name of the corresponding row and column number.
